# Sarcopenia and Cognitive Decline in Older Adults: Targeting the Muscle–Brain Axis

**DOI:** 10.3390/nu15081853

**Published:** 2023-04-12

**Authors:** Beatrice Arosio, Riccardo Calvani, Evelyn Ferri, Hélio José Coelho-Junior, Angelica Carandina, Federica Campanelli, Veronica Ghiglieri, Emanuele Marzetti, Anna Picca

**Affiliations:** 1 Department of Clinical Sciences and Community Health, University of Milan, 20122 Milan, Italy; beatrice.arosio@unimi.it (B.A.); angelica.carandina@unimi.it (A.C.); 2 Fondazione Policlinico Universitario “Agostino Gemelli” IRCCS, 00168 Rome, Italyveronica.ghiglieri@uniroma5.it (V.G.); picca@lum.it (A.P.); 3 Department of Geriatrics and Orthopedics, Università Cattolica del Sacro Cuore, 00168 Rome, Italy; coelhojunior@hotmail.com.br; 4 Fondazione IRCCS Ca’ Granda Ospedale Maggiore Policlinico, 20122 Milan, Italy; evelyn.ferri@policlinico.mi.it; 5 Department of Neuroscience, Università Cattolica del Sacro Cuore, 00168 Rome, Italy; federica.campanelli@unicatt.it; 6 San Raffaele University, 00168 Rome, Italy; 7 Department of Medicine and Surgery, LUM University, 70100 Casamassima, Italy

**Keywords:** brain-derived neurotrophic factor (BDNF), cytokine, cognition, inflammation, mitochondria, myokines, neurotrophins, neuromuscular junction, nutrition, physical performance

## Abstract

Declines in physical performance and cognition are commonly observed in older adults. The geroscience paradigm posits that a set of processes and pathways shared among age-associated conditions may also serve as a molecular explanation for the complex pathophysiology of physical frailty, sarcopenia, and cognitive decline. Mitochondrial dysfunction, inflammation, metabolic alterations, declines in cellular stemness, and altered intracellular signaling have been observed in muscle aging. Neurological factors have also been included among the determinants of sarcopenia. Neuromuscular junctions (NMJs) are synapses bridging nervous and skeletal muscle systems with a relevant role in age-related musculoskeletal derangement. Patterns of circulating metabolic and neurotrophic factors have been associated with physical frailty and sarcopenia. These factors are mostly related to disarrangements in protein-to-energy conversion as well as reduced calorie and protein intake to sustain muscle mass. A link between sarcopenia and cognitive decline in older adults has also been described with a possible role for muscle-derived mediators (i.e., myokines) in mediating muscle–brain crosstalk. Herein, we discuss the main molecular mechanisms and factors involved in the muscle–brain axis and their possible implication in cognitive decline in older adults. An overview of current behavioral strategies that allegedly act on the muscle–brain axis is also provided.

## 1. Introduction

The decline in physical performance during aging contributes to the occurrence of negative health-related events that affects an individual’s health status and quality of life [1,2,3,4,5]. Great attention has been paid to the age-associated declines in muscle mass and strength/function, given their close association with loss of mobility, disability, institutionalization, and mortality [1,3,4,5,6,7], besides being integral to the definition of frailty and sarcopenia [8,9].

A great deal of research has been conducted to identify strategies to avoid, or at least postpone, muscle atrophy and dynapenia during aging [10,11,12]. The practice of specific physical exercise routines and the adherence to some dietary patterns have shown promising results [13,14,15,16,17,18]. In particular, resistance and power training modalities have been shown to successfully improve frailty-related parameters. A high adherence to Mediterranean diet has been associated with better physical performance and cognition in older adults as well as with reduced risk of cognitive decline in non-demented older individuals [13,14,15,16,17,18]. However, an incomplete knowledge of the molecular pathways driving age-related muscle wasting has hampered the identification of targets for drug development.

Through the recognition of aging as a main driver of muscle decline, the geroscience paradigm provides a solid basis for addressing the complex pathophysiology of physical frailty and sarcopenia (PF&S) [19]. Indeed, mitochondrial dysfunction, inflammation, metabolic alterations, declines in cellular stemness, and altered intracellular signaling have all been associated with muscle aging [20,21]. However, aging may not be the only factor contributing to PF&S. For instance, physical inactivity, insufficient protein intake, multimorbidity, and chronic degenerative diseases increase the risk of sarcopenia and frailty [2,22,23,24,25,26,27,28].

Neurological factors also play a role in the pathophysiology of sarcopenia. Neuromuscular junctions (NMJs), while showing the structural features of other chemical synapses, act as a bridge between the nervous (motor neuron) and skeletal muscle (myofiber) systems. NMJs play a relevant role in age-related musculoskeletal impairment [29,30]. Whether NMJ changes trigger or follow the age-associated decline of muscle mass and strength is unsolved.

Specific patterns of circulating levels of inflammatory, hormonal, and neurotrophic factors have been reported in PF&S [31]. The mediators identified are mostly related to metabolic disarrangements as well as failure in protein-to-energy conversion, including an altered ratio between catabolic (e.g., catecholamines, cortisol, glucagon, and pro-atrophy cytokines) and anabolic factors (e.g., growth hormone, insulin, and insulin-like growth factors (IGFs)) [32]. A reduced calorie and protein intake has also been included among the mechanisms involved in muscle wasting in advanced age [32]. Recently, a link between sarcopenia and cognition in older adults has been described, and a possible role for muscle-derived mediators, called myokines, has been proposed in mediating muscle–brain crosstalk [33]. However, the relationship between sarcopenia and cognitive decline as well as the underlying mechanisms remain to be clarified.

Herein, we discuss the main molecular mechanisms and factors known to be involved in the muscle–brain axis and their possible implication in cognitive decline in older adults. Behavioral strategies currently under investigation in this setting are also illustrated.

## 2. Molecular Mechanisms and Mediators of Muscle–Brain Crosstalk

**Figure 1:**
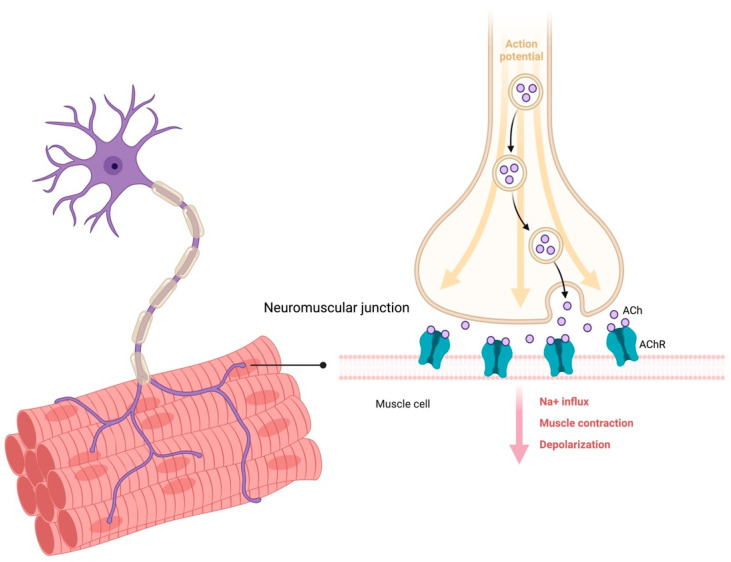
Schematic representation of a neuromuscular junction. Abbreviations: Ach, acetylcholine; AChR, acetylcholine receptor. Created with BioRender.com, accessed on 14 March 2023.

A set of stimuli, including mechanical loading and uptake of calcium during physical exercise and stimulation of insulin, IGF-1, and amino acid signaling, converge on the phosphatidyl inositol 3-kinase (PI3K)–serine/threonine kinase 1 (AKT)– mechanistic target of rapamycin complex 1 (mTORC1) axis and contribute to regulating muscle growth [34,35]. Physical exercise activates PI3K signaling through the integration of mechanical stimuli via integrins onto the skeletal myocyte membrane (sarcolemma) [36]. These signals lead to the phosphorylation of focal adhesion kinases that ultimately activate the PI3K–phosphoinositide-dependent kinase 1 (PDK1)–AKT–mTOR cascade [36]. Besides this local control of muscle growth, specialized and highly orchestrated activities involving communication between nervous and muscular systems are also in place to achieve adequate physical performance levels. The coordination of a movement by the brain requires that the upper motor neurons of the motor cortex signal to the lower motor neurons of the spinal cord posterior area via an action potential. This impulse is transmitted from the motor neuron cell body via its axon to the NMJs. These are composed of presynaptic motor nerve terminals containing synaptic vesicles that carry the neurotransmitter acetylcholine (ACh), synaptic clefts, and the postsynaptic membranes that express Ach receptors (AChRs) (Figure 1). The arrival of an action potential at the presynaptic element opens voltage-dependent calcium channels and leads to calcium-mediated release of ACh in the synaptic cleft. Herein, ACh binds to nicotinic AChRs which, in turn, activate voltage-dependent dihydropyridine receptors in the sarcolemma and ryanodine receptors in the sarcoplasmic reticulum. Schwann cells, a specialized type of glial cells, cover the nerve terminal by forming a basal lamina that merges with the sarcolemma at the boundary of the NMJ [37,38,39]. Fibroblast-like cells, such as keratinocytes or perisynaptic fibroblasts, form a layer over the NMJ and contribute to the repair and regeneration of the nerve [39,40]. It is clear, then, that the maintenance of muscle mass and function requires adequate innervation and regular NMJ activation. Therefore, disarrangements in any of these elements may impact muscle health [29].

During aging, NMJs undergo morphological and functional alterations, including changes in the composition and organization of pre- and postsynaptic membranes, a reduction in the number of neurotransmitter-containing synaptic vesicles, and a slower axonal transport [41]. Similar modifications have been identified in motor neuron diseases (e.g., amyotrophic lateral sclerosis), indicating that strategies aimed at preserving the integrity of NMJs may be crucial for understanding the mechanisms underlying both aging and motor neuron diseases [42,43].

Aging is often accompanied by a loss of muscle mass, strength, and function, referred to as sarcopenia [44,45,46,47,48,49,50]. Sarcopenia is a global health problem [51], with a prevalence ranging from 10 to 40% past the age of 60 [52,53]. The pathophysiology of sarcopenia is still largely unknown, but age-associated impairments of muscle tissue and alterations in neurological factors are implicated in its development [54]. The nervous system, in addition to controlling muscle contraction and voluntary movements [55], is implicated in the determination of myoblast orientation [56], specification of muscle fibers, and regulation of the expression of myosin heavy chain (MHC) isoforms [57]. The question of whether changes in the NMJs trigger or follow sarcopenia remains unsolved. Elevated serum levels of C-terminal agrin fragment (CAF) resulting from NMJ disassembly and denervation are associated with sarcopenia [58], pleading in favor of the hypothesis that the integrity of NMJs is essential for the preservation of both motor nerve and muscle fibers [59]. In animal models, impairment in the expression of the proteoglycan agrin produces phenotypic alterations resembling those observed in aged NMJs and sarcopenia [60]. Moreover, an upregulation of NMJ-associated genes (MuSk and Lrp4 genes) has been observed in both aged and denervated muscles of young mice [61]. Altogether, these findings suggest that aging may cause NMJ deterioration [62].

NMJs and PI3K–AKT–mTOR axis regulation cooperate in the modulation of muscle protein synthesis and growth. The PI3K–AKT–mTOR axis triggers muscle protein synthesis and blunts proteolysis, thus changing muscle protein metabolism towards growth in a balance with autophagy-driven degradation. This modulation seems to occur in conjunction with the stabilization of NMJs, allowing efficient excitation–contraction coupling [63,64,65]. In the setting of aging and associated oxidative stress, the PI3K–AKT–mTORC1 axis can also instigate muscle protein degradation via enhancing AKT signaling [66]. AKT is a serine-threonine protein kinase that regulates cell turnover, proteostasis, and apoptosis, via phosphorylation of transcriptional regulators (e.g., Forkhead box protein O (FoxO)) and downregulation of atrogenes expression [66]. Atrogenes belong to the E3 ubiquitin ligases, a family of enzymes that form multicomplexes and mediate the rate-limiting step of the ubiquitin–proteasome system. This is accomplished via the ubiquitination of substrates intended for degradation. The two best characterized muscle-specific ubiquitin-ligases are muscle RING-finger 1 (MuRF1) and muscle atrophy F-box (Atrogin-1/MAFbx). Other E3 ubiquitin ligases, such as tripartite motif-containing protein 32 (Trim32), are involved in the degradation and renovation of components of the contractile striated muscles units (sarcomeres) [67]. Premature sarcopenia has been identified in knock-out mice for Trim32 [68]. In addition, an adequate Trim32 protein expression has been associated with muscle mass maintenance, muscle reinnervation, and NMJ plasticity during aging in humans [69]. A segmented NMJ morphology and markers of fiber denervation, such as a higher expression of the neural cell adhesion molecule (NCAM), have also been identified in murine models with reduced mTORC1 signaling [65]. Taken as a whole, these findings indicate that NMJ deterioration and an imbalance in PI3K–AKT–mTOR axis may act synergistically to induce age-related muscle decline.

Some neuropsychiatric disorders have also been linked to alterations in the gut microbiota composition and increased intestinal permeability, leading to reduced production of short-chain fatty acids (SCFAs) and enhanced translocation of microbial byproducts into the circulation [70]. Here, endotoxins produced by microorganisms, such as the lipopolysaccharide, contribute to chronic inflammation and insulin resistance, which both promote the development of sarcopenia [71,72]. SCFAs can also bind to skeletal muscle receptors 2 and 3 and induce the release of IGF-1 through pathways that support glucose absorption and metabolism. The PI3K–AKT–mTOR pathway is activated by this protein’s interaction with the insulin receptor substrate 1 (IRS1), which enhances muscle protein synthesis and prevents protein degradation [73]. A reduction in IGF-1 levels and SCFA synthesis has been reported in pathological conditions. For instance, IGF-1 and PI3K–AKT–mTOR pathway suppression, which reduces protein synthesis, has been linked to sarcopenia [74]. Low gastrointestinal levels of SCFAs have been related to increased subclinical chronic inflammation, which is also linked to sarcopenia [75]. Altogether, these findings indicate that the muscle–gut–brain interrelationship may represent an additional level to be considered for the development of interventions targeting the muscle–brain axis.

The skeletal muscle has recently been recognized as a main source of several mediators, collectively known as myokines, that regulate bodily homeostasis and physiological reserves [76,77]. Myokines are a heterogeneous set of biomolecules, including inflammatory cytokines (e.g., interleukin-6 (IL-6), IL-7, IL-8, IL-15), brain-derived neurotrophic factor (BDNF), fibroblast growth factor 21 (FGF21), chemokines, irisin, myostatin, leukemia inhibitory factor (LIF), and secreted protein acidic and rich in cysteine (SPARC) [78,79,80]. Among neurotrophins, BDNF has been associated with the regulation of body metabolism and weight [81]. The protein expression of BDNF is upregulated in muscles after exercise and promotes fatty acid oxidation via the AMP-activated protein kinase (AMPK) pathway [82]. BDNF produced by the muscle also regulates myogenesis, muscle regeneration, and satellite cell activation [83,84]. At the NMJ, BDNF plays a central role in motor neuron viability, enhancement of ACh presynaptic release, and postsynaptic maintenance [85,86,87]. BDNF is secreted as pro-BDNF by both motor neurons and myofibers [88] and is cleaved by extracellular metalloproteases and the tissue-type plasminogen activator (tPA)/plasmin system into its mature form [89]. Evidence suggests that pro- and mature forms of BDNF play opposite functions in NMJ regulation [89]. In particular, pro-BDNF preferentially binds to motor neurons and pan-neurotrophin receptor p75NR in skeletal muscle. This leads to the activation of nuclear factor κB (NF-κB) or c-jun N-terminal kinase (JNK) which both favor the production of inflammatory cytokines by the muscle [90] and neuronal apoptosis [89,91], thus negatively affecting synaptic transmission [89]. The mature form of BDNF is involved in the potentiation of synaptic transmission by preferentially binding to tropomyosin-related receptor kinases (Trk) A and B [89]. Trk receptors are expressed by both motor neurons and skeletal myocytes, and their downstream signaling leads to the activation of PI3K, mitogen-activated protein kinase (MAPK), and phospholipase C-γ (PLC-γ) pathways [89], ultimately supporting neuronal survival [91]. In addition, the activation of PLC-γ in the motor neuron induces PKC-mediated phosphorylation of synaptosomal-associated protein of 25 kDa (SNAP25), a member of the soluble N-ethylmaleimide sensitive factor attachment protein receptor (SNARE) protein complex, and consequent regulation of ACh-vesicle pool refilling [92]. Besides adequate physical exercise, recent studies suggest that dietary supplementation with antioxidants improves muscle health and physical performance [93]. Antioxidants, such as epigallocatechin 3-gallate (EGCG) [94], curcumin [95], and resveratrol [96], could modulate the expression of BDNF. Similarly, BDNF regulators could be affected by antioxidant treatment [97,98].

## 3. Sarcopenia and Cognitive Decline in Older Adults: Myokines at the Interface of the Muscle–Brain Axis

Sarcopenia has a multifactorial pathophysiology, encompassing muscle-specific and non-specific processes (e.g., neuromuscular degeneration, metabolic alterations, and oxidative stress) [99]. Although existing data are mostly associative, there is increasing evidence indicating that the progression of sarcopenia may also contribute to cognitive decline and, perhaps, dementia [100], neuropsychiatric disorders [101], and brain atrophy [102].

The coexistence of sarcopenia and cognitive decline in old age has been widely documented [103], with a direct relationship between reduced gait and poor cognitive performance [104,105,106,107,108,109]. A greater muscle fitness has also been shown to predict changes in brain structure and cognitive function over 10 years in community-dwelling older female twins, regardless of lifestyle and health-related factors [110]. However, little is known about the temporal relationship between muscle wasting and cognitive impairment.

Vascular dysfunction, metabolic derangements (e.g., insulin resistance, high blood cholesterol), and physical inactivity are amongst the major risk factors for cognitive decline [111,112,113]. Increasing evidence highlights a strong link between sarcopenia and cognitive decline that may converge on myokine secretion as a key molecular mechanism [33].

Myokines, cytokines, and chemokines produced and released by skeletal myocytes are involved in multiple physiological and pathological functions and preserve systemic homeostasis. These proteins are secreted into the circulation by myotubes and can hold both pro- and anti-inflammatory signaling roles, such as IL-6, IL-7, IL-8, IL-15, BDNF, angiopoietin-like 4, myostatin, irisin, and gamma-amino isobutyric acid [114]. Furthermore, they can mediate inter-organ signaling, particularly muscle–brain crosstalk, to support global cognitive function and higher complex functions, like learning, memory, and motor coordination [115,116].

Muscle contraction regulates myokine expression, enriching their levels in the bloodstream. Several studies indicate that myokine release, induced by exercise, mediates a crosstalk between the brain and the muscle [114,117]. Myokine signaling may also explain the beneficial effects of physical activity on cognition in older adults, with an increase in the activity of prefrontal cortex and hippocampus, two brain regions implicated in memory and cognition [116,118,119,120,121]. The engagement in community-based exercise programs has shown to ameliorate physical function, cognition, and independence in older adults with Alzheimer’s disease [122]. Furthermore, results from a systematic review and meta-analysis showed that physical activity could reduce the risk of incident vascular dementia by 30–40% in active older adults compared with physically inactive peers [123]. Longitudinal observational studies indicated a lower risk of cognitive decline and dementia in physically active older people [124]. Finally, neuroimaging studies have shown an association between cognitive performance and gait control. Indeed, cognitive therapy has been shown to be successful at preventing falls, and walking programs have been reported to reduce the risk of dementia. Accordingly, individuals with cognitive impairment show slower gait speed compared with the control group [125].

**Figure 2:**
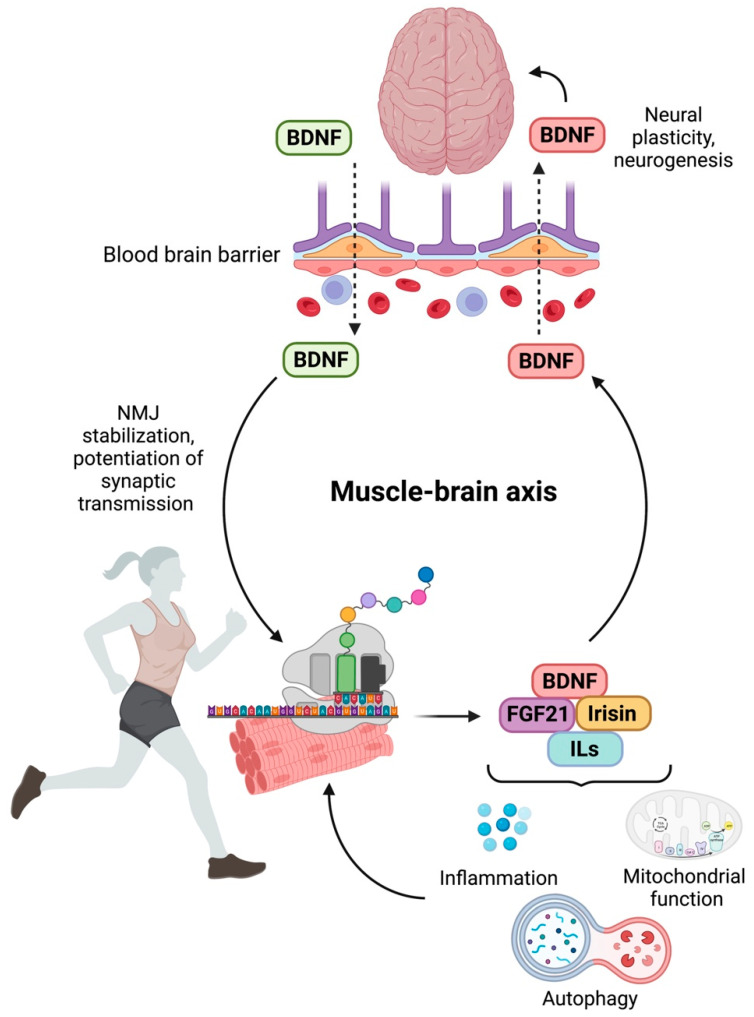
Schematic representation of the muscle–brain axis. Abbreviations: BDNF, brain-derived neurotrophic factor; FGF21, fibroblast growth factor 21; ILs, interleukins; NMJ, neuromuscular junction. Created with BioRender.com, accessed on 14 March 2023.

On the other hand, a detrimental effect of a physically inactive lifestyle has long been established. Physical inactivity is associated with an abnormal synthesis and production of myokines, leading to cognitive deficit and neurogenerative events [126], suggesting a direct impact of muscle contraction on brain health. The loss of skeletal muscle mass and function, typical of sarcopenia, has been associated with an imbalance in myokine secretion [54,116], with possible adverse effects on brain function. The reduced release of myokines promotes and accelerates the progression of changes in the central nervous system (CNS) that lead to cognitive decline [127,128,129]. Epidemiological studies have demonstrated that these alterations are also part of the signaling pathways driving sarcopenia and cognitive impairment [54,129,130]. For instance, the myokine irisin, upon binding to its neuronal receptor, stimulates BDNF expression and enhances cognition (Figure 2). Hence, BDNF produced in the brain following physical activity may protect from cognitive decline through irisin-induced signaling mechanisms [131,132]. The identification of the pathophysiological mechanisms underlying sarcopenia is essential to gain insights into the relationship between muscular dysfunction and cognitive impairment that can be exploited to improve non-pharmacological interventions.

## 4. Targeting the Muscle–Brain Axis: Current Strategies and Emerging Targets

Sarcopenia, the progressive decline of muscle mass and strength/function, is a frequent corollary of the aging process and leads to multiple adverse health-related outcomes, ultimately impacting mobility and the ability to perform daily activities [133]. Given the increasing proportion of older adults worldwide, interventions to counteract sarcopenia are urgently needed.

To date, the most effective approaches are those targeting lifestyle habits, including the promotion of physical activity and quality of diet. A reduction in general activities in advanced age has been indicated as a main contributor to the onset of sarcopenia [134]. Instability of NMJs and increased serum levels of markers associated with NMJ deterioration (e.g., CAF) have been observed during long periods of inactivity, for instance in the setting of bed rest and space flight, as well as in the context of sarcopenia [135,136]. Exercise conveys its beneficial effects on muscle by modulating pathways involved in fiber type composition, muscle growth, and NMJ remodeling [137,138]. Furthermore, a regular physical activity, and, in particular, aerobic exercise, can prevent mitochondrial dysfunction and oxidative damage at the level of motor neurons and NMJs [139,140]. Aerobic exercise also seems to convey beneficial effects by maintaining an adequate release of neurotrophins (e.g., BDNF) that preserve the neuromuscular system [140].

Another aspect that makes physical activity necessary to maintain homeostatic controls of multiple functions concerns the actions of exercise-induced cytokines on gastrointestinal hormones. Muscle-derived IL-6 released during exercise is paralleled by a systemic IL-6 increase in mice [141]. IL-6 triggers the release of glucagon-like peptide 1 (GLP-1) by intestinal cells and subsequently modulates hunger through CNS appetite control systems. As an effect of exercise, irisin levels also increase dramatically in healthy young individuals [142]. Injections of irisin in rats boost ghrelin concentrations and food intake. Muscle-derived irisin has been implicated in the induction of ghrelin release from the gastrointestinal system and hunger modulation in the CNS [143]. An increase in the levels of N-lactoyl-phenylalanine, which reduces appetite and obesity risk, has also been reported during exercise [144]. Further to this, plasma concentrations of peptide YY (PYY) and pancreatic polypeptide (PP) increase during physical activity and can induce a rapid suppression of appetite [145,146,147]. Therefore, elevated levels of PYY, GLP-1, and PP during exercise may be linked to the so-called “exercise-induced anorexia” [145]. However, the mechanisms by which cytokines secreted by the muscle modulate intestinal endocrine cells are unclear, and additional research is warranted to clarify the molecular mechanisms underlying their mutual influence.

In addition to the effects of exercise on the gastrointestinal system, several pieces of evidence also indicate that dietary interventions, for instance, supplementation with antioxidants, can improve muscle health and physical performance [148]. The beneficial effects of dietary antioxidants in maintaining NMJ integrity during aging are not completely clear. However, several natural compounds have been indicated to potentially have such an effect.

For instance, a deficiency of vitamin D, a secosteroid hormone involved in the modulation of several physiological processes, alters the expression of genes and proteins involved in NMJ integrity in an experimental mouse model [149]. Vitamin D supplementation combined with adequate physical exercise reduced CAF concentration in pre-frail community older adults, thus indicating a potentially positive effect of this vitamin on NMJs [150]. Recently, a working group of the European Society for Clinical and Economic Aspects of Osteoporosis, Osteoarthritis and Musculoskeletal Diseases (ESCEO) has recognized the importance of adequate vitamin D supplementation in patients at high risk of vitamin D deficiency as well as for the management of musculoskeletal disease and for reducing the risk of fractures, falls, or osteoarthritis [151].

Beneficial effects on skeletal muscle mass and bone density have also been reported for different isoforms of vitamin E [152]. Moreover, low systemic levels of vitamin E have been associated with a higher prevalence and risk of frailty in older adults [153,154,155,156]. A combined supplementation of vitamin D and vitamin E has been shown to be even more effective at improving muscle mass and strength in sarcopenic older adults [157]. As a matter of fact, both vitamins possess antioxidant and anti-inflammatory properties and contrast cognitive impairment and neurodegeneration in old age [158,159]. Indeed, in a cohort of community-dwelling older adults, a significant association has been described between low vitamin D levels and worse cognitive performance, independent of the presence/absence of cognitive impairment [158]. A similar association was found between levels of different isoforms of vitamin E and the risk of Alzheimer’s disease [159].

In addition to vitamins, dietary protein has been reported to play a vital role in maintaining muscle homeostasis. High-quality protein sources such as fish, lean meat, non-fat dairy and dairy-like products, and soy might be preferable choices to counteract sarcopenia. While numerous studies indicate significant associations between protein intake and physical performance in older adults, investigations on protein consumption and cognitive performance have provided mixed results [160]. A recent systematic review found no significant association between protein intake and global cognitive function in older adults [160]. However, significant positive associations between a high protein intake and the function of several cognitive domains (i.e., memory, processing speed, sustained attention, visuospatial, and verbal fluency) have been reported [160].

It has recently been shown that a diet enriched in free essential amino acids combined with resistance exercise training can improve the synthesis of myofibrillar protein, preservation of both NMJs and fiber type, and biogenesis of mitochondria in an experimental animal model [161]. Dietary supplementation with milk fat globular membrane, containing unique polar lipids and membrane-specific proteins, combined with running produces beneficial effects on NMJs in senescence-accelerated mice [162].

Some of the above-mentioned non-pharmacological interventions have reached the clinical stage and multicomponent strategies specifically targeting lifestyle and nutrition have shown success against age-related physical and cognitive decline [163,164]. The results of a randomized controlled trial indicate that physical activity with technological support and nutritional counseling reduced the incidence of mobility disability in older adults with PF&S [163]. Likewise, multidomain lifestyle interventions, including regular health and nutritional guidance, physical and cognitive training, and vascular risk management convey beneficial effects to older adults at risk of cognitive decline and dementia, regardless of their baseline characteristics [164].

## 5. Conclusions

The recognition of a muscle–brain axis and its possible role at the crossroad of sarcopenia and cognitive impairment may shed new light for the identification of novel therapeutics for managing physical and cognitive frailty in older adults. Targeting the muscle–brain axis may positively impact the quality of life of older adults and of those with neurodegenerative disease conditions, ultimately reducing direct and indirect healthcare costs. However, additional studies integrating clinical and biological readouts via multi-marker and multi-platform strategies are needed to clarify the molecular mechanisms involved in the muscle–brain axis and identify those that may be exploited for drug-developing purposes. As of today, only lifestyle interventions have shown success at managing sarcopenia in older adults, while pharmacological treatment options are missing. Ongoing investigations indicate a beneficial effect of angiotensin-converting enzyme 1 inhibitors and angiotensin II receptor blockers (e.g., losartan), widely used hypertensive drugs, in the maintenance of muscle mass. These drugs can modulate muscle catabolic and anabolic pathways by regulating protein turnover, apoptosis, and collagen metabolism [165,166,167,168]. Some studies also indicate that antihypertensive drugs can reduce the risk of cognitive impairment and slow the rate of cognitive decline in older adults with Alzheimer’s disease regardless of the presence of hypertension [169]. Multi-omics and integrative network analyses have identified a map of highly correlated metabolites and tissue-specific genes in preclinical models treated with modulators of the renin-angiotensin system as a remedy against declines in physical and cognitive function [170]. More conclusive data in humans from large and well characterized cohorts of older adults are highly sought after.

## Data Availability

Not applicable.

## References

[1] Lauretani F., Russo C.R., Bandinelli S., Bartali B., Cavazzini C., Di Iorio A., Corsi A.M., Rantanen T., Guralnik J.M., Ferrucci L. (2003). Age-associated changes in skeletal muscles and their effect on mobility: An operational diagnosis of sarcopenia. J. Appl. Physiol..

[2] Pavasini R., Guralnik J., Brown J.C., di Bari M., Cesari M., Landi F., Vaes B., Legrand D., Verghese J., Wang C. (2016). Short physical performance battery and all-cause mortality: Systematic review and meta-analysis. BMC Med..

[3] Suzuki T., Bean J.F., Fielding R.A. (2001). Muscle power of the ankle flexors predicts functional performance in community-dwelling older women. J. Am. Geriatr. Soc..

[4] Bean J.F., Kiely D.K., Larose S., Goldstein R., Frontera W.R., Leveille S.G. (2010). Are changes in leg power responsible for clinically meaningful improvements in mobility in older adults?. J. Am. Geriatr. Soc..

[5] Guralnik J.M., Simonsick E.M., Ferrucci L., Glynn R.J., Berkman L.F., Blazer D.G., Scherr P.A., Wallace R.B. (1994). A short physical performance battery assessing lower extremity function: Association with self-reported disability and prediction of mortality and nursing home admission. J. Gerontol..

[6] Janssen I., Baumgartner R.N., Ross R., Rosenberg I.H., Roubenoff R. (2004). Skeletal muscle cutpoints associated with elevated physical disability risk in older men and women. Am. J. Epidemiol..

[7] Newman A.B., Kupelian V., Visser M., Simonsick E., Goodpaster B., Nevitt M., Kritchevsky S.B., Tylavsky F.A., Rubin S.M., Harris T.B. (2003). Sarcopenia: Alternative definitions and associations with lower extremity function. J. Am. Geriatr. Soc..

[8] Fried L.P., Tangen C.M., Walston J., Newman A.B., Hirsch C., Gottdiener J., Seeman T., Tracy R., Kop W.J., Burke G. (2001). Frailty in older adults: Evidence for a phenotype. J. Gerontol. A Biol. Sci. Med. Sci..

[9] Cruz-Jentoft A.J., Bahat G.G., Bauer J.J., Boirie Y., Bruyère O., Cederholm T., Cooper C., Landi F., Rolland Y., Sayer A.A. (2019). Sarcopenia: Revised European consensus on definition and diagnosis. Age Ageing.

[10] Cruz-Jentoft A.J., Landi F., Schneider S.M., Zúñiga C., Arai H., Boirie Y., Chen L.-K., Fielding R.A., Martin F.C., Michel J.-P. (2014). Prevalence of and interventions for sarcopenia in ageing adults: A systematic review. Report of the International Sarcopenia Initiative (EWGSOP and IWGS). Age Ageing.

[11] Dent E., Martin F.C., Bergman H., Woo J., Romero-Ortuno R., Walston J.D. (2019). Management of frailty: Opportunities, challenges, and future directions. Lancet.

[12] Cruz-Jentoft A.J., Sayer A.A. (2019). Sarcopenia. Lancet.

[13] Coelho-Júnior H.J., Uchida M.C., Picca A., Bernabei R., Landi F., Calvani R., Cesari M., Marzetti E. (2021). Evidence-based recommendations for resistance and power training to prevent frailty in community-dwellers. Aging Clin. Exp. Res..

[14] Coelho-Júnior H.J., Trichopoulou A., Panza F. (2021). Cross-sectional and longitudinal associations between adherence to Mediterranean diet with physical performance and cognitive function in older adults: A systematic review and meta-analysis. Ageing Res. Rev..

[15] Cacciatore S., Calvani R., Marzetti E., Picca A., Coelho-Júnior H.J., Martone A.M., Massaro C., Tosato M., Landi F. (2023). Low adherence to mediterranean diet is associated with probable sarcopenia in community-dwelling older adults: Results from the Longevity Check-Up (Lookup) 7+ Project. Nutrients.

[16] Izquierdo M., Merchant R.A., Morley J.E., Anker S.D., Aprahamian I., Arai H., Aubertin-Leheudre M., Bernabei R., Cadore E.L., Cesari M. (2021). International exercise recommendations in older adults (ICFSR): Expert consensus guidelines. J. Nutr. Health Aging.

[17] Martone A.M.A.M., Marzetti E., Calvani R., Picca A., Tosato M., Santoro L., Di Giorgio A., Nesci A., Sisto A., Santoliquido A. (2017). Exercise and protein intake: A synergistic approach against sarcopenia. BioMed Res. Int..

[18] Coelho-Júnior H.J., Calvani R., Tosato M., Landi F., Picca A., Marzetti E. (2022). Protein intake and physical function in older adults: A systematic review and meta-analysis. Ageing Res. Rev..

[19] Schmauck-Medina T., Molière A., Lautrup S., Zhang J., Chlopicki S., Madsen H.B., Cao S., Soendenbroe C., Mansell E., Vestergaard M.B. (2022). New hallmarks of ageing: A 2022 Copenhagen ageing meeting summary. Aging.

[20] Marzetti E., Guerra F., Calvani R., Marini F., Biancolillo A., Gervasoni J., Primiano A., Coelho-Júnior H.J., Landi F., Bernabei R. (2020). Circulating mitochondrial-derived vesicles, inflammatory biomarkers and amino acids in older adults with physical frailty and sarcopenia: A Preliminary BIOSPHERE multi-marker study using sequential and orthogonalized covariance selection–Linear discriminant analysis. Front. Cell Dev. Biol..

[21] Muñoz-Cánoves P., Neves J., Sousa-Victor P. (2020). Understanding muscle regenerative decline with aging: New approaches to bring back youthfulness to aged stem cells. FEBS J..

[22] Chang M., Cohen-Mansfield J., Ferrucci L., Leveille S., Volpato S., De Rekeneire N., Guralnik J.M. (2004). Incidence of loss of ability to walk 400 meters in a functionally limited older population. J. Am. Geriatr. Soc..

[23] Fanning J., Rejeski W.J., Chen S.H., Guralnik J., Pahor M., Miller M.E. (2020). Relationships between profiles of physical activity and major mobility disability in the LIFE Study. J. Am. Geriatr. Soc..

[24] Pahor M., Guralnik J.M., Anton S.D., Ambrosius W.T., Blair S.N., Church T.S., Espeland M.A., Fielding R.A., Gill T.M., Glynn N.W. (2020). Impact and lessons from the lifestyle interventions and independence for elders (LIFE) clinical trials of physical activity to prevent mobility disability. J. Am. Geriatr. Soc..

[25] Groessl E.J., Kaplan R.M., Rejeski W.J., Katula J.A., Glynn N.W., King A.C., Anton S.D., Walkup M., Lu C.J., Reid K. (2019). Physical activity and performance impact long-term quality of life in older adults at risk for major mobility disability. Am. J. Prev. Med..

[26] Guralnik J.M., Ferrucci L., Simonsick E.M., Salive M.E., Wallace R.B. (1995). Lower-extremity function in persons over the age of 70 years as a predictor of subsequent disability. N. Engl. J. Med..

[27] Veronese N., Smith L., Cereda E., Maggi S., Barbagallo M., Dominguez L.J., Koyanagi A. (2021). Multimorbidity increases the risk for sarcopenia onset: Longitudinal analyses from the English Longitudinal Study of Ageing. Exp. Gerontol..

[28] Lim H.-S., Park Y.-H., Suh K., Yoo M.H., Park H.K., Kim H.J., Lee J.-H., Byun D.-W. (2018). Association between sarcopenia, sarcopenic obesity, and chronic disease in Korean elderly. J. Bone Metab..

[29] Casati M., Costa A.S., Capitanio D., Ponzoni L., Ferri E., Agostini S., Lori E. (2019). The biological foundations of sarcopenia: Established and promising markers. Front. Med..

[30] Gonzalez-Freire M., de Cabo R., Studenski S.A., Ferrucci L. (2014). The neuromuscular junction: Aging at the crossroad between nerves and muscle. Front. Aging Neurosci..

[31] Calvani R., Picca A., Marini F., Biancolillo A., Gervasoni J., Persichilli S., Primiano A., Coelho-Junior H.J., Cesari M., Bossola M. (2020). Identification of biomarkers for physical frailty and sarcopenia through a new multi-marker approach: Results from the BIOSPHERE study. GeroScience.

[32] Pasini E., Corsetti G., Aquilani R., Romano C., Picca A., Calvani R., Dioguardi F.S. (2018). Protein-amino acid metabolism disarrangements: The hidden enemy of chronic age-related conditions. Nutrients.

[33] Scisciola L., Fontanella R.A., Surina, Cataldo V., Paolisso G., Barbieri M. (2021). Sarcopenia and cognitive function: Role of myokines in muscle brain cross-talk. Life.

[34] Sartori R., Romanello V., Sandri M. (2021). Mechanisms of muscle atrophy and hypertrophy: Implications in health and disease. Nat. Commun..

[35] Baehr L.M., Hughes D.C., Waddell D.S., Bodine S.C. (2022). SnapShot: Skeletal muscle atrophy. Cell.

[36] Philp A., Hamilton D.L., Baar K. (2011). Highlighted topic signals mediating skeletal muscle remodeling by activity signals mediating skeletal muscle remodeling by resistance exercise: PI3-kinase independent activation of mTORC1. J. Appl. Physiol..

[37] Castro R., Taetzsch T., Vaughan S.K., Godbe K., Chappell J., Settlage R.E., Valdez G. (2020). Specific labeling of synaptic schwann cells reveals unique cellular and molecular features. eLife.

[38] Feng Z., Ko C.P. (2008). Schwann cells promote synaptogenesis at the neuromuscular junction via transforming growth factor-beta1. J. Neurosci..

[39] Sugiura Y., Lin W. (2011). Neuron-glia interactions: The roles of Schwann cells in neuromuscular synapse formation and function. Biosci. Rep..

[40] Court F.A., Gillingwater T.H., Melrose S., Sherman D.L., Greenshields K.N., Morton A.J., Harris J.B., Willison H.J., Ribchester R.R. (2008). Identity, developmental restriction and reactivity of extralaminar cells capping mammalian neuromuscular junctions. J. Cell Sci..

[41] Hickling J.K., Fenton C.M., Howland K., Marsh S.G.E., Rothbard J.B. (1990). Peptides recognized by class I restricted T cells also bind to MHC class II molecules. Int. Immunol..

[42] Pratt J., De Vito G., Narici M., Boreham C. (2021). Neuromuscular junction aging: A role for biomarkers and exercise. J. Gerontol. A Biol. Sci. Med. Sci..

[43] Lepore E., Casola I., Dobrowolny G., Musarò A. (2019). Neuromuscular junction as an entity of nerve-muscle communication. Cells.

[44] Siparsky P.N., Kirkendall D.T., Garrett W.E. (2014). Muscle changes in aging: Understanding sarcopenia. Sports Health.

[45] Vandervoort A.A., Symons T.B. (2001). Functional and metabolic consequences of sarcopenia. Can. J. Appl. Physiol..

[46] Clark B.C. (2019). Neuromuscular changes with aging and sarcopenia. J. Frailty Aging.

[47] Rosenberg I.H. (1997). Sarcopenia: Origins and clinical relevance. J. Nutr..

[48] Baker B.A. (2018). Efficacy of age-specific high-intensity stretch-shortening contractions in reversing dynapenia, sarcopenia, and loss of skeletal muscle quality. J. Funct. Morphol. Kinesiol..

[49] Clark B.C., Manini T.M. (2008). Sarcopenia =/= dynapenia. J. Gerontol. A Biol. Sci. Med. Sci..

[50] Morley J.E., Abbatecola A.M., Argiles J.M., Baracos V., Bauer J., Bhasin S., Cederholm T., Stewart Coats A.J., Cummings S.R., Evans W.J. (2011). Sarcopenia with limited mobility: An international consensus. J. Am. Med. Dir. Assoc..

[51] Shafiee G., Keshtkar A., Soltani A., Ahadi Z., Larijani B., Heshmat R. (2017). Prevalence of sarcopenia in the world: A systematic review and meta-analysis of general population studies. J. Diabetes Metab. Disord..

[52] Cruz-Jentoft A.J., Baeyens J.P., Bauer J.M., Boirie Y., Cederholm T., Landi F., Martin F.C., Michel J.P., Rolland Y., Schneider S.M. (2010). European working group on sarcopenia in older people sarcopenia: European consensus on definition and diagnosis: Report of the European working group on sarcopenia in older people. Age Ageing.

[53] Yazar T., Olgun Yazar H. (2019). Prevalance of sarcopenia according to decade. Clin. Nutr. ESPEN.

[54] Sui S.X., Williams L.J., Holloway-Kew K.L., Hyde N.K., Pasco J.A. (2020). Skeletal muscle health and cognitive function: A narrative review. Int. J. Mol. Sci..

[55] Rudolf R., Khan M.M., Witzemann V. (2019). Motor endplate-anatomical, functional, and molecular concepts in the historical perspective. Cells.

[56] Mège R.M., Goudou D., Giaume C., Nicolet M., Rieger F. (1994). Is intercellular communication via gap junctions required for myoblast fusion?. Cell Adhes. Commun..

[57] Schiaffino S., Reggiani C. (1996). Molecular diversity of myofibrillar proteins: Gene regulation and functional significance. Physiol. Rev..

[58] Landi F., Calvani R., Lorenzi M., Martone A.M., Tosato M., Drey M., D’Angelo E., Capoluongo E., Russo A., Bernabei R. (2016). Serum levels of C-terminal agrin fragment (CAF) are associated with sarcopenia in older multimorbid community-dwellers: Results from the ilSIRENTE study. Exp. Gerontol..

[59] Shigemoto K., Kubo S., Mori S., Yamada S., Akiyoshi T., Miyazaki T. (2010). Muscle weakness and neuromuscular junctions in aging and disease. Geriatr. Gerontol. Int..

[60] Bütikofer L., Zurlinden A., Bolliger M.F., Kunz B., Sonderegger P. (2011). Destabilization of the neuromuscular junction by proteolytic cleavage of agrin results in precocious sarcopenia. FASEB J..

[61] Ibebunjo C., Chick J.M., Kendall T., Eash J.K., Li C., Zhang Y., Vickers C., Wu Z., Clarke B.A., Shi J. (2013). Genomic and proteomic profiling reveals reduced mitochondrial function and disruption of the neuromuscular junction driving rat sarcopenia. Mol. Cell. Biol..

[62] Dalkin W., Taetzsch T., Valdez G. (2016). The fibular nerve injury method: A reliable assay to identify and test factors that repair neuromuscular junctions. J. Vis. Exp..

[63] Vainshtein A., Sandri M. (2020). Signaling pathways that control muscle mass. Int. J. Mol. Sci..

[64] Ham D.J., Börsch A., Lin S., Thürkauf M., Weihrauch M., Reinhard J.R., Delezie J., Battilana F., Wang X., Kaiser M.S. (2020). The neuromuscular junction is a focal point of mTORC1 signaling in sarcopenia. Nat. Commun..

[65] Baraldo M., Geremia A., Pirazzini M., Nogara L., Solagna F., Türk C., Nolte H., Romanello V., Megighian A., Boncompagni S. (2020). Skeletal muscle mTORC1 regulates neuromuscular junction stability. J. Cachexia Sarcopenia Muscle.

[66] Tang H., Inoki K., Brooks S.V., Okazawa H., Lee M., Wang J., Kim M., Kennedy C.L., Macpherson P.C.D., Ji X. (2019). mTORC1 underlies age-related muscle fiber damage and loss by inducing oxidative stress and catabolism. Aging Cell.

[67] Cohen S., Zhai B., Gygi S.P., Goldberg A.L. (2012). Ubiquitylation by Trim32 causes coupled loss of desmin, Z-bands, and thin filaments in muscle atrophy. J. Cell Biol..

[68] Mokhonova E.I., Avliyakulov N.K., Kramerova I., Kudryashova E., Haykinson M.J., Spencer M.J. (2015). The E3 ubiquitin ligase TRIM32 regulates myoblast proliferation by controlling turnover of NDRG2. Hum. Mol. Genet..

[69] Skoglund E., Grönholdt-Klein M., Rullman E., Thornell L.E., Strömberg A., Hedman A., Cederholm T., Ulfhake B., Gustafsson T. (2020). Longitudinal muscle and myocellular changes in community-dwelling men over two decades of successful aging-The ULSAM cohort revisited. J. Gerontol. A Biol. Sci. Med. Sci..

[70] Graziani C., Talocco C., De Sire R., Petito V., Lopetuso L.R., Gervasoni J., Persichilli S., Franceschi F., Ojetti V., Gasbarrini A. (2019). Intestinal permeability in physiological and pathological conditions: Major determinants and assessment modalities. Eur. Rev. Med. Pharmacol. Sci..

[71] Bindels L.B., Delzenne N.M. (2013). Muscle wasting: The gut microbiota as a new therapeutic target?. Int. J. Biochem. Cell. Biol..

[72] Lochlainn M.N., Bowyer R.C.E., Steves C.J. (2018). Dietary protein and muscle in aging people: The potential role of the gut microbiome. Nutrients.

[73] Schiaffino S., Mammucari C. (2011). Regulation of skeletal muscle growth by the IGF1-Akt/PKB pathway: Insights from genetic models. Skelet. Muscle.

[74] Sirago G., Picca A., Calvani R., Coelho-Júnior H.J., Marzetti E. (2022). Mammalian target of rapamycin (MTOR) Signaling at the crossroad of muscle fiber fate in sarcopenia. Int. J. Mol. Sci..

[75] den Besten G., Lange K., Havinga R., van Dijk T.H., Gerding A., van Eunen K., Müller M., Groen A.K., Hooiveld G.J., Bakker B.M. (2013). Gut-derived short-chain fatty acids are vividly assimilated into host carbohydrates and lipids. Am. J. Physiol. Gastrointest. Liver Physiol..

[76] Coelho-Junior H.J., Picca A., Calvani R., Uchida M.C., Marzetti E. (2019). If my muscle could talk: Myokines as a biomarker of frailty. Exp. Gerontol..

[77] Calvani R., Marini F., Cesari M., Tosato M., Anker S.D., von Haehling S., Miller R.R., Bernabei R., Landi F., Marzetti E. (2015). SPRINTT consortium Biomarkers for physical frailty and sarcopenia: State of the science and future developments. J. Cachexia Sarcopenia Muscle.

[78] Pedersen B.K., Febbraio M.A. (2012). Muscles, exercise and obesity: Skeletal muscle as a secretory organ. Nat. Rev. Endocrinol..

[79] Whitham M., Febbraio M.A. (2016). The ever-expanding myokinome: Discovery challenges and therapeutic implications. Nat. Rev. Drug Discov..

[80] Ojima K., Oe M., Nakajima I., Shibata M., Muroya S., Chikuni K., Hattori A., Nishimura T. (2015). The importance of subfragment 2 and C-terminus of myosin heavy chain for thick filament assembly in skeletal muscle cells. Anim. Sci. J..

[81] Briana D.D., Malamitsi-Puchner A. (2018). Developmental origins of adult health and disease: The metabolic role of BDNF from early life to adulthood. Metabolism.

[82] Matthews V.B., Åström M.B., Chan M.H.S., Bruce C.R., Krabbe K.S., Prelovsek O., Åkerström T., Yfanti C., Broholm C., Mortensen O.H. (2009). Brain-derived neurotrophic factor is produced by skeletal muscle cells in response to contraction and enhances fat oxidation via activation of AMP-activated protein kinase. Diabetologia.

[83] Levy M.J.F., Boulle F., Steinbusch H.W., van den Hove D.L.A., Kenis G., Lanfumey L. (2018). Neurotrophic factors and neuroplasticity pathways in the pathophysiology and treatment of depression. Psychopharmacology.

[84] Hempstead B.L., Martin-Zanca D., Kaplan D.R., Parada L.F., Chao M.V. (1991). High-affinity NGF binding requires coexpression of the trk proto-oncogene and the low-affinity NGF receptor. Nature.

[85] Moreira-Pais A., Ferreira R., Oliveira P.A., Duarte J.A. (2022). A neuromuscular perspective of sarcopenia pathogenesis: Deciphering the signaling pathways involved. GeroScience.

[86] Colombo E., Bedogni F., Lorenzetti I., Landsberger N., Previtali S.C., Farina C. (2013). Autocrine and immune cell-derived BDNF in human skeletal muscle: Implications for myogenesis and tissue regeneration. J. Pathol..

[87] Sakuma K., Yamaguchi A. (2011). The recent understanding of the neurotrophin’s role in skeletal muscle adaptation. J. Biomed. Biotechnol..

[88] Leßmann V., Brigadski T. (2009). Mechanisms, locations, and kinetics of synaptic BDNF secretion: An update. Neurosci. Res..

[89] Lu B. (2003). Pro-region of neurotrophins: Role in synaptic modulation. Neuron.

[90] Aby K., Antony R., Eichholz M., Srinivasan R., Li Y. (2021). Enhanced pro-BDNF-p75NTR pathway activity in denervated skeletal muscle. Life Sci..

[91] Diniz C.R.A.F., Casarotto P.C., Resstel L., Joca S.R.L. (2018). Beyond good and evil: A putative continuum-sorting hypothesis for the functional role of proBDNF/BDNF-propeptide/mBDNF in antidepressant treatment. Neurosci. Biobehav. Rev..

[92] Nagy G., Matti U., Nehring R.B., Binz T., Rettig J., Neher E., Sørensen J.B. (2002). Protein kinase C-dependent phosphorylation of synaptosome-associated protein of 25 kDa at Ser187 potentiates vesicle recruitment. J. Neurosci..

[93] Franceschi F., Feregalli B., Togni S., Cornelli U., Giacomelli L., Eggenhoffner R., Belcaro G. (2016). A novel phospholipid delivery system of curcumin (Meriva^®^) preserves muscular mass in healthy aging subjects. Eur. Rev. Med. Pharmacol. Sci..

[94] Abdelmeguid N.E., Hammad T.M., Abdel-Moneim A.M., Salam S.A. (2022). Effect of epigallocatechin-3-gallate on stress-induced depression in a mouse model: Role of interleukin-1β and brain-derived neurotrophic factor. Neurochem. Res..

[95] Jin T., Zhang Y., Botchway B.O.A., Zhang J., Fan R., Zhang Y., Liu X. (2022). Curcumin can improve Parkinson’s disease via activating BDNF/PI3k/Akt signaling pathways. Food Chem. Toxicol..

[96] Sechi S., Chiavolelli F., Spissu N., Di Cerbo A., Canello S., Guidetti G., Fiore F., Cocco R. (2015). An antioxidant dietary supplement improves brain-derived neurotrophic factor levels in serum of aged dogs: Preliminary results. J. Vet. Med..

[97] Zhang M., Xue Y., Chen H., Meng L., Chen B., Gong H., Zhao Y., Qi R. (2019). Resveratrol inhibits MMP3 and MMP9 expression and secretion by suppressing TLR4/NF- κ B/STAT3 activation in Ox-LDL-treated HUVECs. Oxidative Med. Cell. Longev..

[98] Tsantarliotou M.P., Lavrentiadou S.N., Psalla D.A., Margaritis I.E., Kritsepi M.G., Zervos I.A., Latsari M.I., Sapanidou V.G., Taitzoglou I.A., Sinakos Z.M. (2019). Suppression of plasminogen activator inhibitor-1 (PAI-1) activity by crocin ameliorates lipopolysaccharide-induced thrombosis in rats. Food Chem. Toxicol..

[99] Picca A., Calvani R., Sirago G., Coelho-Junior H.J., Marzetti E. (2021). Molecular routes to sarcopenia and biomarker development: Per aspera ad astra. Curr. Opin. Pharmacol..

[100] Ogawa Y., Kaneko Y., Sato T., Shimizu S., Kanetaka H., Hanyu H. (2018). Sarcopenia and muscle functions at various stages of Alzheimer disease. Front. Neurol..

[101] Szlejf C., Suemoto C.K., Brunoni A.R., Viana M.C., Moreno A.B., Matos S.M.A., Lotufo P.A., Benseñor I.M. (2019). Depression is associated with sarcopenia due to low muscle strength: Results from the ELSA-Brasil study. J. Am. Med. Dir. Assoc..

[102] Tanabe C., Reed M.J., Pham T.N., Penn K., Bentov I., Kaplan S.J. (2019). Association of brain atrophy and masseter sarcopenia with 1-year mortality in older trauma patients. JAMA Surg..

[103] Peng T.C., Chen W.L., Wu L.W., Chang Y.W., Kao T.W. (2020). Sarcopenia and cognitive impairment: A systematic review and meta-analysis. Clin. Nutr..

[104] Wu Y.H., Liu L.K., Chen W.T., Lee W.J., Peng L.N., Wang P.N., Chen L.K. (2015). Cognitive function in individuals with physical frailty but without dementia or cognitive complaints: Results from the I-Lan longitudinal aging study. J. Am. Med. Dir. Assoc..

[105] Callisaya M.L., Blizzard C.L., Wood A.G., Thrift A.G., Wardill T., Srikanth V.K. (2015). Longitudinal relationships between cognitive decline and gait slowing: The Tasmanian study of cognition and gait. J. Gerontol. A Biol. Sci. Med. Sci..

[106] Mielke M.M., Roberts R.O., Savica R., Cha R., Drubach D.I., Christianson T., Pankratz V.S., Geda Y.E., Machulda M.M., Ivnik R.J. (2013). Assessing the temporal relationship between cognition and gait: Slow gait predicts cognitive decline in the Mayo Clinic study of aging. J. Gerontol. A Biol. Sci. Med. Sci..

[107] Callisaya M.L., Beare R., Phan T.G., Blizzard L., Thrift A.G., Chen J., Srikanth V.K. (2013). Brain structural change and gait decline: A longitudinal population-based study. J. Am. Geriatr. Soc..

[108] Taylor M.E., Lasschuit D.A., Lord S.R., Delbaere K., Kurrle S.E., Mikolaizak A.S., Kvelde T., Close J.C.T. (2017). Slow gait speed is associated with executive function decline in older people with mild to moderate dementia: A one year longitudinal study. Arch. Gerontol. Geriatr..

[109] Watson N.L., Rosano C., Boudreau R.M., Simonsick E.M., Ferrucci L., Sutton-Tyrrell K., Hardy S.E., Atkinson H.H., Yaffe K., Satterfield S. (2010). Executive function, memory, and gait speed decline in well-functioning older adults. J. Gerontol. A Biol. Sci. Med. Sci..

[110] Steves C.J., Mehta M.M., Jackson S.H.D., Spector T.D. (2016). Kicking back cognitive ageing: Leg power predicts cognitive ageing after ten years in older female twins. Gerontology.

[111] Kong S.H., Park Y.J., Lee J.Y., Cho N.H., Moon M.K. (2018). Insulin resistance is associated with cognitive decline among older Koreans with normal baseline cognitive function: A prospective community-based cohort study. Sci. Rep..

[112] Yang F.N., Stanford M., Jiang X. (2020). Low cholesterol level linked to reduced semantic fluency performance and reduced gray matter volume in the medial temporal lobe. Front. Aging Neurosci..

[113] Law C.K., Lam F.M., Chung R.C., Pang M.Y. (2020). Physical exercise attenuates cognitive decline and reduces behavioural problems in people with mild cognitive impairment and dementia: A systematic review. J. Physiother..

[114] Severinsen M.C.K., Pedersen B.K. (2020). Muscle-organ crosstalk: The emerging roles of myokines. Endocr. Rev..

[115] Pedersen B.K. (2019). Physical activity and muscle-brain crosstalk. Nat. Rev. Endocrinol..

[116] Kim S., Choi J.Y., Moon S., Park D.H., Kwak H.B., Kang J.H. (2019). Roles of myokines in exercise-induced improvement of neuropsychiatric function. Pflugers Arch..

[117] Chen W., Wang L., You W., Shan T. (2021). Myokines mediate the cross talk between skeletal muscle and other organs. J. Cell. Physiol..

[118] Colcombe S.J., Erickson K.I., Scalf P.E., Kim J.S., Prakash R., McAuley E., Elavsky S., Marquez D.X., Hu L., Kramer A.F. (2006). Aerobic exercise training increases brain volume in aging humans. J. Gerontol. A Biol. Sci. Med. Sci..

[119] Erickson K.I., Prakash R.S., Voss M.W., Chaddock L., Hu L., Morris K.S., White S.M., Wójcicki T.R., McAuley E., Kramer A.F. (2009). Aerobic fitness is associated with hippocampal volume in elderly humans. Hippocampus.

[120] Kramer A.F., Colcombe S. (2018). Fitness effects on the cognitive function of older adults: A meta-analytic study-revisited. Perspect. Psychol. Sci..

[121] Voss M.W., Erickson K.I., Prakash R.S., Chaddock L., Kim J.S., Alves H., Szabo A., Phillips S.M., Wójcicki T.R., Mailey E.L. (2013). Neurobiological markers of exercise-related brain plasticity in older adults. Brain. Behav. Immun..

[122] Vreugdenhil A., Cannell J., Davies A., Razay G. (2012). A community-based exercise programme to improve functional ability in people with Alzheimer’s disease: A randomized controlled trial. Scand. J. Caring Sci..

[123] Aarsland D., Sardahaee F.S., Anderssen S., Ballard C. (2010). Is physical activity a potential preventive factor for vascular dementia? A systematic review. Aging Ment. Health.

[124] Blondell S.J., Hammersley-Mather R., Veerman J.L. (2014). Does physical activity prevent cognitive decline and dementia?: A systematic review and meta-analysis of longitudinal studies. BMC Public Health.

[125] Amboni M., Barone P., Hausdorff J.M. (2013). Cognitive contributions to gait and falls: Evidence and implications. Mov. Disord..

[126] Iizuka K., Machida T., Hirafuji M. (2014). Skeletal muscle is an endocrine organ. J. Pharmacol. Sci..

[127] Yu L., Boyle P.A., Leurgans S.E., Wilson R.S., Bennett D.A., Buchman A.S. (2019). Incident mobility disability, mild cognitive impairment, and mortality in community-dwelling older adults. Neuroepidemiology.

[128] Boyle P.A., Buchman A.S., Wilson R.S., Leurgans S.E., Bennett D.A. (2009). Association of muscle strength with the risk of Alzheimer disease and the rate of cognitive decline in community-dwelling older persons. Arch. Neurol..

[129] Beeri M.S., Leugrans S.E., Delbono O., Bennett D.A., Buchman A.S. (2021). Sarcopenia is associated with incident Alzheimer’s dementia, mild cognitive impairment, and cognitive decline. J. Am. Geriatr. Soc..

[130] Aprahamian I., Cipolli G.C., Yassuda M.S. (2020). Sarcopenia and cognitive impairment: Possible physiopathological causation or just a spurious association?. Clin. Nutr..

[131] Lourenco M.V., Frozza R.L., de Freitas G.B., Zhang H., Kincheski G.C., Ribeiro F.C., Gonçalves R.A., Clarke J.R., Beckman D., Staniszewski A. (2019). Exercise-linked FNDC5/irisin rescues synaptic plasticity and memory defects in Alzheimer’s models. Nat. Med..

[132] de Freitas G.B., Lourenco M.V., De Felice F.G. (2020). Protective actions of exercise-related FNDC5/Irisin in memory and Alzheimer’s disease. J. Neurochem..

[133] Visser M., Goodpaster B.H., Kritchevsky S.B., Newman A.B., Nevitt M., Rubin S.M., Simonsick E.M., Harris T.B. (2005). Muscle mass, muscle strength, and muscle fat infiltration as predictors of incident mobility limitations in well-functioning older persons. J. Gerontol. A Biol. Sci. Med. Sci..

[134] Berger M.J., Doherty T.J. (2010). Sarcopenia: Prevalence, mechanisms, and functional consequences. Interdiscip. Top. Gerontol..

[135] Harvey J.A., Chastin S.F.M., Skelton D.A. (2013). Prevalence of sedentary behavior in older adults: A systematic review. Int. J. Environ. Res. Public Health.

[136] Yang W., Zhang H. (2016). Effects of hindlimb unloading on neurotrophins in the rat spinal cord and soleus muscle. Brain Res..

[137] Mammucari C., Milan G., Romanello V., Masiero E., Rudolf R., Del Piccolo P., Burden S.J., Di Lisi R., Sandri C., Zhao J. (2007). FoxO3 controls autophagy in skeletal muscle in vivo. Cell Metab..

[138] Zampieri S., Mammucari C., Romanello V., Barberi L., Pietrangelo L., Fusella A., Mosole S., Gherardi G., Höfer C., Löfler S. (2016). Physical exercise in aging human skeletal muscle increases mitochondrial calcium uniporter expression levels and affects mitochondria dynamics. Physiol. Rep..

[139] Joseph A.M., Adhihetty P.J., Leeuwenburgh C. (2016). Beneficial effects of exercise on age-related mitochondrial dysfunction and oxidative stress in skeletal muscle. J. Physiol..

[140] Nishimune H., Stanford J.A., Mori Y. (2014). Role of exercise in maintaining the integrity of the neuromuscular junction. Muscle Nerve.

[141] Ellingsgaard H., Hauselmann I., Schuler B., Habib A.M., Baggio L.L., Meier D.T., Eppler E., Bouzakri K., Wueest S., Muller Y.D. (2011). Interleukin-6 enhances insulin secretion by increasing glucagon-like peptide-1 secretion from L cells and alpha cells. Nat. Med..

[142] Anastasilakis A.D., Polyzos S.A., Saridakis Z.G., Kynigopoulos G., Skouvaklidou E.C., Molyvas D., Vasiloglou M.F., Apostolou A., Karagiozoglou-Lampoudi T., Siopi A. (2014). Circulating irisin in healthy, young individuals: Day-night rhythm, effects of food intake and exercise, and associations with gender, physical activity, diet, and body composition. J. Clin. Endocrinol. Metab..

[143] Tekin S., Erden Y., Ozyalin F., Cigremis Y., Colak C., Sandal S. (2017). The effects of intracerebroventricular infusion of irisin on feeding behaviour in rats. Neurosci. Lett..

[144] Li V.L., He Y., Contrepois K., Liu H., Kim J.T., Wiggenhorn A.L., Tanzo J.T., Tung A.S.H., Lyu X., Zushin P.J.H. (2022). An exercise-inducible metabolite that suppresses feeding and obesity. Nature.

[145] Martins C., Morgan L.M., Bloom S.R., Robertson M.D. (2007). Effects of exercise on gut peptides, energy intake and appetite. J. Endocrinol..

[146] Ueda S.Y., Yoshikawa T., Katsura Y., Usui T., Fujimoto S. (2009). Comparable effects of moderate intensity exercise on changes in anorectic gut hormone levels and energy intake to high intensity exercise. J. Endocrinol..

[147] Ueda S.Y., Yoshikawa T., Katsura Y., Usui T., Nakao H., Fujimoto S. (2009). Changes in gut hormone levels and negative energy balance during aerobic exercise in obese young males. J. Endocrinol..

[148] Bates C.J., Prentice A., Cole T.J., Van Der Pols J.C., Doyle W., Finch S., Smithers G., Clarke P.C. (1999). Micronutrients: Highlights and research challenges from the 1994-5 national diet and nutrition survey of people aged 65 years and over. Br. J. Nutr..

[149] Scicchitano B.M., Pelosi L., Sica G., Musarò A. (2018). The physiopathologic role of oxidative stress in skeletal muscle. Mech. Ageing Dev..

[150] Drey M., Sieber C.C., Bauer J.M., Uter W., Dahinden P., Fariello R.G., Vrijbloed J.W. (2013). C-terminal Agrin fragment as a potential marker for sarcopenia caused by degeneration of the neuromuscular junction. Exp. Gerontol..

[151] Chevalley T., Brandi M.L., Cashman K.D., Cavalier E., Harvey N.C., Maggi S., Cooper C., Al-Daghri N., Bock O., Bruyère O. (2022). Role of vitamin D supplementation in the management of musculoskeletal diseases: Update from an European Society of Clinical and Economical Aspects of Osteoporosis, Osteoarthritis and Musculoskeletal Diseases (ESCEO) working group. Aging Clin. Exp. Res..

[152] Mulligan A.A., Hayhoe R.P.G., Luben R.N., Welch A.A. (2021). Positive associations of dietary intake and plasma concentrations of vitamin E with skeletal muscle mass, heel bone ultrasound attenuation and fracture risk in the EPIC-Norfolk cohort. Antioxidants.

[153] Pilleron S., Weber D., Pérès K., Colpo M., Gomez-Cabrero D., Stuetz W., Dartigues J.-F., Ferrucci L., Bandinelli S., Garcia-Garcia F.J. (2019). Patterns of circulating fat-soluble vitamins and carotenoids and risk of frailty in four European cohorts of older adults. Eur. J. Nutr..

[154] Kochlik B., Stuetz W., Pérès K., Pilleron S., Féart C., García García F.J., Bandinelli S., Gomez-Cabrero D., Rodriguez-Mañas L., Grune T. (2019). Associations of fat-soluble micronutrients and redox biomarkers with frailty status in the FRAILOMIC initiative. J. Cachexia Sarcopenia Muscle.

[155] Ble A., Cherubini A., Volpato S., Bartali B., Walston J.D., Windham B.G., Bandinelli S., Lauretani F., Guralnik J.M., Ferrucci L. (2006). Lower plasma vitamin E levels are associated with the frailty syndrome: The InCHIANTI study. J. Gerontol. A Biol. Sci. Med. Sci..

[156] Bartali B., Frongillo E.A., Bandinelli S., Lauretani F., Semba R.D., Fried L.P., Ferrucci L. (2006). Low nutrient intake is an essential component of frailty in older persons. J. Gerontol. A Biol. Sci. Med. Sci..

[157] Bo Y., Liu C., Ji Z., Yang R., An Q., Zhang X., You J., Duan D., Sun Y., Zhu Y. (2019). A high whey protein, vitamin D and E supplement preserves muscle mass, strength, and quality of life in sarcopenic older adults: A double-blind randomized controlled trial. Clin. Nutr..

[158] Arosio B., Rossi P.D., Ferri E., Cesari M., Vitale G. (2022). Characterization of vitamin D status in older persons with cognitive impairment. Nutrients.

[159] Casati M., Boccardi V., Ferri E., Bertagnoli L., Bastiani P., Ciccone S., Mansi M., Scamosci M., Rossi P.D., Mecocci P. (2020). Vitamin E and Alzheimer’s disease: The mediating role of cellular aging. Aging Clin. Exp. Res..

[160] Coelho-Júnior H.J., Calvani R., Landi F., Picca A., Marzetti E. (2021). Protein intake and cognitive function in older adults: A systematic review and meta-analysis. Nutr. Metab. Insights.

[161] Jang J., Koh J.H., Kim Y., Kim H.J., Park S., Chang Y., Jung J., Wolfe R.R., Kim I.Y. (2022). Balanced free essential amino acids and resistance exercise training synergistically improve dexamethasone-induced impairments in muscle strength, endurance, and insulin sensitivity in mice. Int. J. Mol. Sci..

[162] Yano M., Haramizu S., Ota N., Minegishi Y., Shimotoyodome A. (2019). Continuous supplementation of milk fat globule membrane with habitual exercise from a young age improves motor coordination and skeletal muscle function in aged mice. J. Nutr. Sci. Vitaminol..

[163] Bernabei R., Landi F., Calvani R., Cesari M., Del Signore S., Anker S.D., Bejuit R., Bordes P., Cherubini A., Cruz-Jentoft A.J. (2022). Multicomponent intervention to prevent mobility disability in frail older adults: Randomised controlled trial (SPRINTT project). BMJ.

[164] Rosenberg A., Ngandu T., Rusanen M., Antikainen R., Bäckman L., Havulinna S., Hänninen T., Laatikainen T., Lehtisalo J., Levälahti E. (2018). Multidomain lifestyle intervention benefits a large elderly population at risk for cognitive decline and dementia regardless of baseline characteristics: The FINGER trial. Alzheimers. Dement..

[165] Song Y.H., Li Y., Du J., Mitch W.E., Rosenthal N., Delafontaine P. (2005). Muscle-specific expression of IGF-1 blocks angiotensin II-induced skeletal muscle wasting. J. Clin. Investig..

[166] Burks T.N., Andres-Mateos E., Marx R., Mejias R., Van Erp C., Simmers J.L., Walston J.D., Ward C.W., Cohn R.D. (2011). Losartan restores skeletal muscle remodeling and protects against disuse atrophy in sarcopenia. Sci. Transl. Med..

[167] Cabello-Verrugio C., Acuña M.J., Morales M.G., Becerra A., Simon F., Brandan E. (2011). Fibrotic response induced by angiotensin-II requires NAD(P)H oxidase-induced reactive oxygen species (ROS) in skeletal muscle cells. Biochem. Biophys. Res. Commun..

[168] Cabello-Verrugio C., Morales M.G., Rivera J.C., Cabrera D., Simon F. (2015). Renin-angiotensin system: An old player with novel functions in skeletal muscle. Med. Res. Rev..

[169] Soto M.E., Abellan Van Kan G., Nourhashemi F., Gillette-Guyonnet S., Cesari M., Cantet C., Rolland Y., Vellas B. (2013). Angiotensin-converting enzyme inhibitors and Alzheimer’s disease progression in older adults: Results from the Réseau sur la Maladie d’Alzheimer Français cohort. J. Am. Geriatr. Soc..

[170] Baptista L.C., Zumbro E.L., Graham Z.A., Hernandez A.R., Buchanan T., Sun Y., Yang Y., Banerjee A., Verma A., Li Q. (2023). Multi-omics profiling of the impact of an angiotensin (1-7)-expressing probiotic combined with exercise training in aged male rats. J. Appl. Physiol. (1985).

